# Supplementary material for: Emergence and Spread of Piscine orthoreovirus Genotype 3
Source: Pathogens. 2020 Oct 7;9(10):823. doi: 10.3390/pathogens9100823 (PMC7601675; doi:10.3390/pathogens9100823)
Supplement: Supplementary file 1 [file pathogens-09-00823-s001.zip › Table S1.docx]

**Table S1**: Ct values of pooled strong positive, weak positive, suspect, and negative samples of all tested dilutions (v:v).

| **Pool** | **Dilution** | **Ct value (dRn)** |
| --- | --- | --- |
| Strong positive | Undiluted | 17.62 |
|  | 1:1 | 17.94 |
|  | 1:2 | 18.57 |
|  | 1:4 | 19.21 |
|  | 1:6 | 19.53 |
|  | 1:9 | 20.42 |
| Weak positive | Undiluted | 33.96 |
|  | 1:1 | 35.20 |
|  | 1:2 | 35.40 |
|  | 1:4 | 36.94 |
|  | 1:6 | 36.46 |
|  | 1:9 | 36.74 |
| Suspect | Undiluted | 35.93 |
|  | 1:1 | No Ct |
|  | 1:2 | 37.27 |
|  | 1:4 | No Ct |
|  | 1:6 | 35.86 |
|  | 1:9 | 36.86 |
| Negative | Undiluted | No Ct |
