# Supplementary material for: Emergence and Spread of Piscine orthoreovirus Genotype 3
Source: Pathogens. 2020 Oct 7;9(10):823. doi: 10.3390/pathogens9100823 (PMC7601675; doi:10.3390/pathogens9100823)
Supplement: Supplementary file 1 [file pathogens-09-00823-s001.zip › Table S2_v2.docx]

|  | Isolate | Country | Year | Species | Disease | GenBank ID |
| --- | --- | --- | --- | --- | --- | --- |
| PRV-3 | DK/95-8109 | Denmark | 1995 | *O. mykiss* |  | L1: MW012884, L2: MW012883,  L3: MW012882, M1: MW012881,  M2: MW012880, M3: MW012879,  S1: MW012878, S2: MW012877,  S3: MW012876, S4: MW012875 |
|  | DK/95-8194 | Denmark | 1995 | *O. mykiss* |  | S1: MT991697 |
|  | DK/95-8183 | Denmark | 1995 | *O. mykiss* |  | S1: MT991681 |
|  | DK/95-8161 | Denmark | 1995 | *O. mykiss* |  | S1: MT991674 |
|  | DK/95-8202 | Denmark | 1995 | *O. mykiss* |  | S1: MT991677 |
|  | DK/95-8201 | Denmark | 1995 | *O. mykiss* |  | S1: MT991675 |
|  | DK/95-8252 | Denmark | 1995 | *O. mykiss* |  | S1: MT991693 |
|  | DK/95-8199 | Denmark | 1995 | *O. mykiss* |  | S1: MT991676, M2: MT991733 |
|  | DK/17-18918-1 | Denmark | 2017 | *O. mykiss* | Yes | S1: MG983785 |
|  | DK/17-18918-6 | Denmark | 2017 | *O. mykiss* | Yes | S1: MG983786 |
|  | DK/17-18918-13 | Denmark | 2017 | *O. mykiss* | Yes | S1: MG983782 |
|  | DK/17-20001-9 | Denmark | 2017 | *O. mykiss* | Yes | S1: MT991704 |
|  | DK/17-20264-3 | Denmark | 2017 | *O. mykiss* | Yes | S1: MT991678, M2: MT991730 |
|  | DK/17-20348-4 | Denmark | 2017 | *O. mykiss* | Yes | S1: MT991682 |
|  | DK/PRV317 | Denmark | 2017 | *O. mykiss* | Yes | L1: MW012874, L2: MW012873,  L3: MW012872, M1: MW012871,  M2: MW012870, M3: MW012869,  S1: MW012868, S2: MW012867,  S3: MW012866, S4: MW012865 |
|  | DK/18-239-6 | Denmark | 2018 | *O. mykiss* | Yes | S1: MT991712 |
|  | DK/18-240-20 | Denmark | 2018 | *O. mykiss* | Yes | S1: MT991713, M2: MT991726 |
|  | DK/18-307-10 | Denmark | 2018 | *O. mykiss* | Yes | S1: MT991684 |
|  | DK/18-357-4 | Denmark | 2018 | *O. mykiss* | Yes | S1: MT991698, M2: MT991725 |
|  | DK/18-358-3 | Denmark | 2018 | *O. mykiss* | Yes | S1: MT991699 |
|  | DK/18-359-3 | Denmark | 2018 | *O. mykiss* | Yes | S1: MT991685 |
|  | DK/18-974-10 | Denmark | 2018 | *O. mykiss* |  | S1: MT991696 |
|  | DK/18-1548-9 | Denmark | 2018 | *O. mykiss* |  | S1: MT991679, M2: MT991743 |
|  | DK/18-1724-24 | Denmark | 2018 | *O. mykiss* |  | S1: MT991680, M2: MT991723 |
|  | DK/18-1725-16 | Denmark | 2018 | *O. mykiss* |  | S1: MT991711, M2: MT991735 |
|  | DK/18-1766-19 | Denmark | 2018 | *O. mykiss* |  | S1: MT991700, M2: MT991732 |
|  | DK/18-2214-6 | Denmark | 2018 | *O. mykiss* |  | S1: MT991683, M2: MT991744 |
|  | DK/18-2235-6 | Denmark | 2018 | *O. mykiss* |  | S1: MT991694, M2: MT991724 |
|  | DK/PRV315 | Denmark | 2018 | *O. mykiss* | Yes | L1: MW012864, L2: MW012863,  L3: MW012862, M1: MW012861,  M2: MW012860, M3: MW012859,  S1: MW012858, S2: MW012857,  S3: MW012856, S4: MW012855 |
|  | DK/18-3758-33 | Denmark | 2018 | *O. mykiss* |  | S1: MT991701, M2: MT991737 |
|  | DK/18-4056-15H | Denmark | 2018 | *O. mykiss* |  | S1: MT991686, M2: MT991729 |
|  | DK/18-4163-36 | Denmark | 2018 | *O. mykiss* |  | S1: MT991691 |
|  | DK/18-4260-7 | Denmark | 2018 | *O. mykiss* | Yes | S1: MT991703, M2: MT991738 |
|  | DK/18-4929-33 | Denmark | 2018 | *O. mykiss* |  | S1: MT991718, M2: MT991742 |
|  | DK/18-5020-3 | Denmark | 2018 | *S. trutta fario* |  | S1: MT991719 |
|  | DK/18-5577-34 | Denmark | 2018 | *O. mykiss* |  | S1: MT991714, M2: MT991752 |
|  | DK/18-5610-33 | Denmark | 2018 | *O. mykiss* |  | S1: MT991689 |
|  | DK/18-5621-13 | Denmark | 2018 | *O. mykiss* |  | S1: MT991695, M2: MT991740 |
|  | DK/18-5649-35 | Denmark | 2018 | *O. mykiss* | Yes | S1: MT991715, M2: MT991734 |
|  | DK/18-5650-58 | Denmark | 2018 | *O. mykiss* |  | S1: MT991702, M2: MT991739 |
|  | DK/18-5888-25 | Denmark | 2018 | *S. trutta fario* |  | S1: MT991716, M2: MT991747 |
|  | DK/18-6340-33 | Denmark | 2018 | *S. trutta fario* |  | S1: MT991720, M2: MT991746 |
|  | DK/18-6518-30 | Denmark | 2018 | *O. mykiss* |  | S1: MT991721, M2: MT991741 |
|  | DK/18-7143-36 | Denmark | 2018 | *O. mykiss* |  | S1: MT991690, M2: MT991731 |
|  | DK/18-7924-33 | Denmark | 2018 | *O. mykiss* |  | S1: MT991687, M2: MT991722 |
|  | DK/18-10460-31 | Denmark | 2018 | *O. mykiss* |  | S1: MT991705, M2: MT991728 |
|  | DK/18-11362-34 | Denmark | 2018 | *O. mykiss* |  | S1: MT991717, M2: MT991745 |
|  | DK/18-16648-32 | Denmark | 2018 | *O. mykiss* |  | S1: MT991688, M2: MT991727 |
|  | DK/19-6546-32 | Denmark | 2019 | *O. mykiss* |  | S1: MT991692, M2: MT991736 |
|  | IT/17-19266-27 | Italy | 2017 | *S. trutta fario* |  | S1: MG983783 |
|  | IT/17-19266-35 | Italy | 2017 | *S. trutta fario* |  | S1: MG983784 |
|  | VT12202013-CGA-2013-3 | Chile | 2013 | *O. kisutch* |  | S1: KU131595 |
|  | VT12202013-CGA-2013-5 | Chile | 2013 | *O. kisutch* |  | S1: KU131596 |
|  | C10/P1.1 | Chile | 2014 | *O. mykiss* |  | S1: KX844965 |
|  | C10/P1.2 | Chile | 2014 | *O. mykiss* |  | S1: KX844964 |
|  | C10/P2.2 | Chile | 2014 | *O. mykiss* |  | S1: KX844962 |
|  | C10/P3.1 | Chile | 2014 | *O. mykiss* |  | S1: KX844961 |
|  | C10/P3.2 | Chile | 2014 | *O. mykiss* |  | S1: KX844960 |
|  | C10/P4.1 | Chile | 2014 | *O. mykiss* |  | S1: KX844951 |
|  | C10/P4.2 | Chile | 2014 | *O. mykiss* |  | S1: KX844959 |
|  | ADLPRV3 | Chile | 2017 | *O. kisutch* |  | L1: MH229776; L2: MH229777;  L3: MH229778; M1: MH229779;  M2: MH229780; M3: MH229781;  S1: MH229785; S2: MH229783; S3: MH229784; S4: MH229782 |
|  | DH/PRV-3 (Kuehn et al) | Germany | 2008 | *S. trutta fario* |  | S1: MH513870; M2: MH513868 |
|  | DH/PRV-3 Salmo trutta | Germany | 2008 | *S. trutta fario* |  | L1: MK286551; L3: MK286550;  M1: MH513866; M2: MK286552;  M3: MK286553; S1: MK286554;  S2: MK286555; S3: MK286556;  S4: MK286557 |
|  | DH/747072017 | Germany | 2017 | *S. salar* |  | S1: MG983787 |
|  | NOR/060214 | Norway | 2013 | *O. mykiss* |  | L1: MG253807; L2: MG253808;  L3: MG253809; M1: MG253810;  M2: MG253811; M3: MG253812;  S1: MG253816; S2: MG253813; S3: MG253814; S4: MG253815 |
|  | 2016-02-100_VY-NOK 31 | Norway | 2016 | *O. mykiss* |  | S1: MT991710, M2: MT991749 |
|  | 2016-02-766_VY-OK 474 | Norway | 2016 | *O. mykiss* |  | S1: MT991706 |
|  | 2016-02-467_VY-OK 368 | Norway | 2016 | *O. mykiss* |  | S1: MT991707, M2: MT991750 |
|  | 2016-02-388_VY-OK 334 | Norway | 2016 | *O. mykiss* |  | S1: MT991708, M2: MT991751 |
|  | 2016-02-214_VY-144 | Norway | 2016 | *O. mykiss* |  | S1: MT991709, M2: MT991748 |
| Out-group | B7274 | Canada | 2013 | *S. salar* |  | L1: KX851978; L2: KX851980;  L3: KX851982; M1: KX851976;  M2: KX851974; M3: KX851972; S1: KX851971; S2: KX851968;  S3: KX851966; S4: KX851964 |
|  | WSKFH12_14 | Canada | 2015 | *O. kisutch* |  | L1: KT429752; L2: KT429751;  L3: KT429750; M1: KT429753;  M2: KT429754; M3: KT429755; S1: KT429756; S2: KT429757;  S3: KT429758; S4: KT429759 |
|  | NOR-1988 | Norway | 1988 | *S. salar* |  | L1: MK675862; L2: MK675863;  L3: MK675864; M1: MK675865;  M2: MK675866; M3: MK675867 S1: MK675868; S2: MK675869;  S3: MK675870; S4: K675871 |
|  | Salmo/GP_2010/NOR | Norway | 2010 | *S. salar* |  | L1: GU994015; L2: GU994014;  L3: GU994013; M1: GU994017;  M2: GU994016; M3: GU994018;  S1: GU994022; S2: GU994019; S3: GU994020; S4: GU994021 |
|  | NOR2012_V3621 | Norway | 2012 | *S. salar* |  | L1: KY429943; L2: KY429944;  L3: KY429945; M1: KY429946;  M2: KY429947; M3: KY429948;  S1: KY429949; S2: KY429950;  S3: KY429951; S4: KY429952 |
|  | PRV-2 | Japan | 2012 | *O. kisutch* |  | L1: LC145609; L2: LC145608;  L3: LC145610; M1: LC145612;  M2: LC145613; M3: LC145611;  S1: LC145616; S2: LC145614;  S3: LC145615; S4:LC145617 |
