# Supplementary material for: Emergence and Spread of Piscine orthoreovirus Genotype 3
Source: Pathogens. 2020 Oct 7;9(10):823. doi: 10.3390/pathogens9100823 (PMC7601675; doi:10.3390/pathogens9100823)
Supplement: Supplementary file 1 [file pathogens-09-00823-s001.zip › Table S3_v2.docx]

**Table S3**: Haplotypes of segment S1

| Haplotype name | Isolates | Time span | Distribution | Species |
| --- | --- | --- | --- | --- |
| DK1 | DK/18-5020-3  DK/18-6340-33 | 2018 | Denmark | *S. trutta fario* |
| DK2 | DK/240-20  DK/5577-34 | 2018 | Denmark | *O. mykiss* |
| DK3 | DK/19-3758-33  DK/18-4260-7  DK/18-1766-19  DK/18-5650-58 | 2018-2019 | Denmark | *O. mykiss* |
| DK4 | DK/PRV317  DK/PRV315  DK/95-8161  DK/95-8201  DK/95-8199  DK/95-8202  DK/17-20264-3  DK/18-1548-9  DK/18-1724-24  DK/95-8183  DK/17-20348-4  DK/18-2214-6  DK/18-307-10  DK/18-359-3  DK/18-4056-15H  DK/18-7924-33  DK/18-16648-32  DK/18-5610-33  DK/18-7143-36  DK/18-4163-36  DK/17-18918-1  DK/17-18918-6  IT/17-19266-35  IT/17-19266-27  DK/95-8252  DK/18-2235-6  DK/18-5621-13  DK/18-357-4  DK/18-358-3  DH/747072017  DK/18-1725-16  DK/18-11362-34 | 1995-2018 | Denmark and Italy | *O. mykiss* and *S. trutta fario* |
| DK5 | DK/18-239-6  DK/95-8194  DK/18-5649-35 | 1995-2018 | Denmark | *O. mykiss* |
| DK6 | DK/17-20001-9  DK/17-18918-13 | 2017 | Denmark | *O. mykiss* |
| DK7 | DK/18-974-10 | 2018 | Denmark | *O. mykiss* |
| DK8 | DK/95-8109 (full genome available) | 1995 | Denmark | *O. mykiss* |
| DK9 | DK/19-6546-32 | 2019 | Denmark | *O. mykiss* |
| DK10 | DK/18-10460-31 | 2018 | Denmark | *O. mykiss* |
| DK11 | DK/18-5888-25 | 2018 | Denmark | *S. trutta fario* |
| DK12 | DK/18-4929-33  DK/18-6518-30 | 2018 | Denmark | *O. mykiss* |
| SCT1 | G1491 | 2017 | Scotland | *O. mykiss* |
| DH1 | DH/PRV-3 (Kuehn et al)  DH/PRV-3 *Salmo trutta* | 2008 | Germany | *S. salar* |
| CH1 | ADLPRV3  C10/P3.1  C10/P2.2  C10/P3.2  VT12202013-CGA-2013-3 | 2013-2017 | Chile | *O. kisutch* and *O. mykiss* |
| CH2 | VT12202013-CGA-2013-5 | 2013 | Chile | *O. kisutch* |
| CH3 | C10/P1.1 | 2014 | Chile | *O. mykiss* |
| CH4 | C10/P4.2 | 2014 | Chile | *O. mykiss* |
| CH5 | C10/P4.1 | 2014 | Chile | *O. mykiss* |
| CH6 | C10/P1.2 | 2014 | Chile | *O. mykiss* |
| NOR1 | NOR/060214 | 2013 | Norway | *O. mykiss* |
| NOR2 | 2016-02-467 VY- OK 368  2016-02-766 VY- OK 474  2016-02-388 VY- OK 334  2016-02-214 VY- 144  2016-02-100 VY- NOK 31 | 2016 | Norway | *O. mykiss* |
