# Supplementary material for: Emergence and Spread of Piscine orthoreovirus Genotype 3
Source: Pathogens. 2020 Oct 7;9(10):823. doi: 10.3390/pathogens9100823 (PMC7601675; doi:10.3390/pathogens9100823)
Supplement: Supplementary file 1 [file pathogens-09-00823-s001.zip › Table S4.docx]

**Tables S4**: Total number of reads and coverage mapping to PRV-3 isolates.

| Segments | DK/18-3659-17 | | DK/18-3659-15 | | DK/95-8109 | |
| --- | --- | --- | --- | --- | --- | --- |
|  | Reads count | Average coverage | Reads count | Average coverage | Reads count | Average coverage |
| L1 | 91,427 | 2,294 | 146,511 | 3,679 | 230,767 | 9,665 |
| L2 | 81,473 | 2,067 | 134,223 | 3,407 | 150,051 | 6,791 |
| L3 | 67,956 | 1,722 | 120,316 | 3,050 | 213,602 | 8,959 |
| M1 | 29,605 | 1,258 | 31,594 | 1,343 | 35,919 | 2,570 |
| M2 | 22,405 | 1,030 | 32,824 | 1,510 | 78,678 | 5,759 |
| M3 | 62,431 | 2,057 | 75,207 | 2,556 | 119,266 | 7,792 |
| S1 | 10,673 | 957 | 13,609 | 1,219 | 49,155 | 6,314 |
| S2 | 14,852 | 1,110 | 14,351 | 1,071 | 47,675 | 5,030 |
| S3 | 12,349 | 1,097 | 12,312 | 1,092 | 32,994 | 4,420 |
| S4 | 7,027 | 673 | 8,862 | 848 | 8,828 | 1,169 |
